# Triptolide Suppresses Glomerular Mesangial Cell Proliferation in Diabetic Nephropathy Is Associated with Inhibition of PDK1/Akt/mTOR Pathway: Erratum

**DOI:** 10.7150/ijbs.101944

**Published:** 2024-09-05

**Authors:** Fei Han, Mei Xue, Yunpeng Chang, Xiaoyu Li, Yang Yang, Bei Sun, Liming Chen

**Affiliations:** Key Laboratory of Hormones and Development (Ministry of Health), Tianjin Key Laboratory of Metabolic Diseases, Tianjin Metabolic Diseases Hospital & Tianjin Institute of Endocrinology, Tianjin Medical University

In our previous paper, an error occurred during the preparation of the IF staining of PCNA in the mannitol group (Fig. 4G), where an incorrect image was used, and the original image was inadvertently omitted. We wish to clarify that this correction does not impact the results of our study as reported in the published paper, and maintain full confidence in the quality of the experiments and the conclusions drawn in the paper.

We deeply regret the oversight that led to this error prior to manuscript submission, which was unfortunately not detected during subsequent review and publication stages. We sincerely apologize for any inconvenience this may have caused to our readers.

Figure 4G should be corrected as follows.

## Figures and Tables

**Figure 4:**
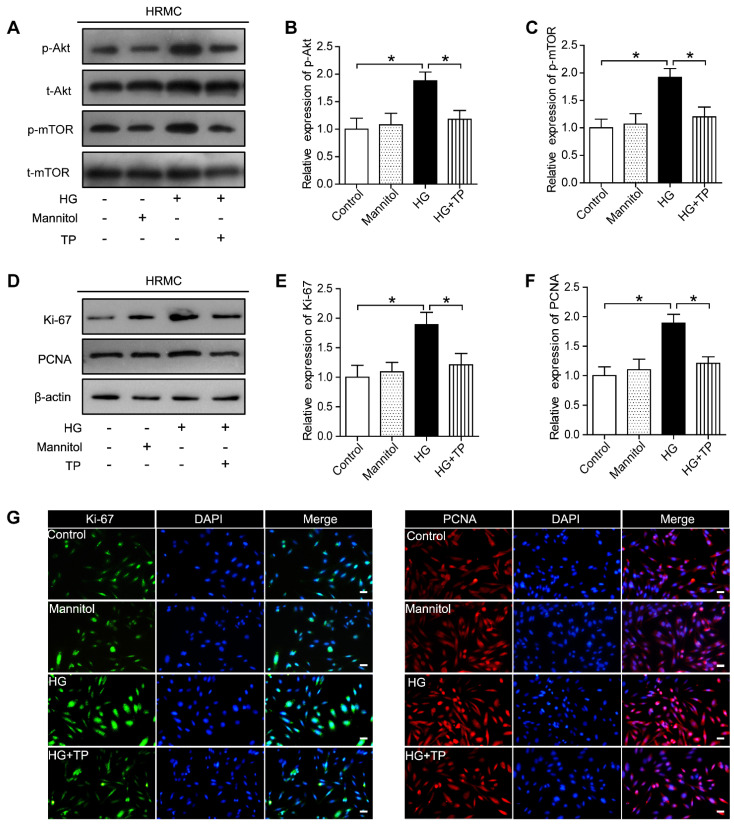
**The effect of TP on proliferating cell markers in HG-treated HRMCs.** (A) Western blot images of Akt/mTOR pathway. (B) Quantification of protein expression of phosphorylation-Akt. (C) Quantification of protein expression of phosphorylation-mTOR. (D) Western blot images of Ki-67 and PCNA. (E) Quantification of Ki-67 protein expression. (F) Quantification of PCNA protein expression. (G) Immunofluorescence images of Ki-67 and PCNA. The scale bar represents 10 μm. Data were reported as mean ± S.D. *P < 0.05

